# Supplementary material for: Thresholds of temperature change for mass extinctions
Source: Nat Commun. 2021 Aug 4;12:4694. doi: 10.1038/s41467-021-25019-2 (PMC8338942; doi:10.1038/s41467-021-25019-2)
Supplement: Supplementary file 1 — Supplementary Information [file 41467_2021_25019_MOESM1_ESM.pdf]

Supplementary Information for

Thresholds of temperature change for mass extinctions

Haijun Song, David B. Kemp, Li Tian, Daoliang Chu, Huyue Song, Xu Dai

Correspondence to: haijunsong@cug.edu.cn

This PDF file includes:

Supplementary Methods and Data
Supplementary Figures 1 to 6
Supplementary Tables 1 to 3

Supplementary Methods and Data

Temperature data

All data for calculating the magnitudes and rates of temperature change are listed in Supplementary Data 1.

Or5, Hirnantian (443.14 - 442.67 Ma)

The magnitude and duration of Hirnantian cooling are from North American carbonate clumped isotope data of Finnegan et al. [1] and the U-Pb dates of Ling et al. [2]. Conodont oxygen isotope data from Canada suggest a consistent cooling trend [3]. The cooling event occurs from the middle of the *Belonechitina gamachiana* chitinozoan Zone to *Spinachitina taugourdeaui* chitinozoan Zone [1,4]. The trend line of Δ47-derived near-surface ocean temperature suggests a temperature drop of ~8.4 °C during the early Hirnantian cooling event (Fig. 3A in ref. [1]). The U-Pb dates of Ling et al. suggest the duration of this cooling event is ~227 kyr [2].

Silurian

The magnitude and duration of temperature change of three bins (i.e., S1, Llandovery; S2, Wenlock and Ludlow; and S3, Pridoli) are from high-resolution conodont oxygen isotope records in the Canadian Arctic Laurentian margin and Baltic craton [5]. Ages are constrained from the Geological Time Scale 2012 [6]. The oxygen isotope value of Silurian seawater was set to -1‰ (VSMOW) [7] since no continental ice sheet has been reported in the Silurian [8] (see Methods in main text). The trends of oxygen isotope change in the Laurentian margin and Baltic craton regions are nearly synchronous, suggesting a global trend that was not significantly affected by local salinity effects.

S1, Llandovery (442.67-433.4 Ma). Conodont oxygen isotopes from both Canadian Arctic and Baltic craton indicate a temperature increase in the early Llandovery followed by a more significant fall in in the late Llandovery [5]. The temperature increase in the Canadian Arctic is ~4.1 °C, which is a little larger than that found in the Baltic craton.

S2, Wenlock and Ludlow (433.4-423 Ma). Conodont oxygen isotope data from the Baltic craton suggest two climate cooling events during the Wenlock and Ludlow interval [5]. The larger one occurred in the early Wenlock with a magnitude of 4.3 °C in 1.4 Myr.

S3, Pridoli (423-419.2 Ma). Conodont oxygen isotopes from the Baltic craton indicate a rapid climb of temperature during the middle Pridoli [5]. Calculated sea surface temperatures increased ~2.6 °C in ~0.9 Myr.

Early-Middle Devonian

The temperature change in three bins (i.e., D1, Lochkovian; D2, Pragian and Emsian; and D3, Eifelian and Givetian) derive from high-resolution conodont oxygen isotope records from Europe (France, Germany, and Czech Republic) and Australia [9]. Ages are from the Geological Time Scale 2012 [10]. The oxygen isotope value of Early-Middle Devonian seawater was set to -1‰ (VSMOW) to reflect an ice-free world in this interval [7] [8]. The similar long-term $\delta^{18}O$ trend in Europe and Australia suggests that the reconstructed temperature curves represent global climatic change rather than local changes.

D1, Lochkovian and Pragian (419.2-407.6 Ma). Conodont oxygen isotopes from Australia suggest a rapid decline of temperature in the Pragian [9]. Sea surface temperature decreased ~7.9 °C in ~4.3 Myr.

D2, Emsian (407.6-393.3 Ma). Conodont oxygen isotope data from Europe suggest a warming event in the late Emsian with a magnitude of ~3.7 °C and a duration of 3.44 Myr [9]. This warming event was followed by a quick rebound in the end-Emsian.

D3, Eifelian and Givetian (393.3-382.7 Ma). Conodont oxygen isotope data from Europe suggests three temperature troughs in the Middle Devonian [9]. The largest magnitude temperature change in this interval was a 5.7 °C decline during the Givetian, with a duration of 2.61 Myr.

Late Devonian

The magnitude and duration of temperature change of two bins (i.e., D4, Frasnian; and D5, Famennian) are from high-resolution conodont oxygen isotope records from South China [11]. Ages derive from the Geological Time Scale 2012 [10]. Only a minor ice age was identified in the late Famennian (Strunian)[12]. We assumed -1‰ (VSMOW) as the oxygen isotope value of seawater for the ice-free world[7] during the Frasnian and early-middle Famennian.

D4, Frasnian (382.7-372.2 Ma). Conodont oxygen isotopes from South China suggest multiple cooling and warming events during the Frasnian[11]. The largest magnitude of climate change was a cooling event that occurred at the end-Frasnian. Sea surface temperatures decreased ~5.2 °C in ~90 kyr.

D5, Famennian (372.2-358.9 Ma). Conodont oxygen isotopes from South China suggests a significant increase of sea surface temperature with a magnitude of ~4.3 °C, over an interval of ~780 kyr.

Carboniferous-Early and Middle Permian

The temperature change of five time bins in the Carboniferous (i.e., C1, Tournaisian; C2, Visean and Serpukhovian; C3, Bashkirian; C4, Moscovian and Kasimovian; C5, Gzhelian) and three time bins in the Early and Middle Permian (i.e., P1, Asselian and Sakmarian; P2, Artinskian; P3, Kungurian and Roadian) derive from conodont oxygen isotope records in Euramerica [13], and South China [14]. Ages are from the Geological Time Scale 2012 [15,16]. The trends in oxygen isotopes in these regions are near-synchronous, suggesting a global pattern. The Late Paleozoic Ice Age (LPIA) was the longest ice age in the Phanerozoic [8,17]. We assumed that the oxygen isotope ratio of seawater during the Pennsylvanian glacial maximum is the same as the Pleistocene Last Glacial Maximum, i.e., +1‰ (VSMOW) [18] because the areas of ice cover in these two intervals were nearly the same [19]. Seawater $\delta^{18}O$ in the intervals between ice-free periods and the Pennsylvanian glacial maximum was set to values between -1‰ and +1‰ (VSMOW) based on available glaciation records during the Carboniferous and Permian [17,19,20].

The Tournaisian and Visean have been recognized as the initial stage of the Late Paleozoic Ice Age, while the Serpukhovian and Early Bashkirian were the major phase of ice sheet expansion [19,20]. Ice sheets began to shrink from the Gzhelian to Capitanian [19,20]. Here, we assumed seawater $\delta^{18}O$ in the initial Tournaisian, the initial Visean, the initial Serpukhovian, the initial Bashkirian, and the mid-Bashkirian were -1‰, -0.5‰, 0‰, 0.5‰, and 1‰ (VSMOW), respectively. The seawater $\delta^{18}O$ in the initial Gzhelian, the initial Asselian, the initial Artinskian, the initial Kungurian, the initial Wordian, and the end-Capitanian were assumed to be 1‰, 0.5‰, 0‰, -0.3‰, -0.7‰, and -1‰ (VSMOW), respectively.

C1, Tournaisian (358.9-346.7 Ma). Conodont oxygen isotope records from Europe (French, Poland, and Spain)[13] show that there was a significant increase of conodont $\delta^{18}O$. Given that the Tournaisian has been recognized as the initial stage of the Late Paleozoic Ice Age [19,20], the shift in conodont $\delta^{18}O$ suggests a cooling event with a magnitude of ~4.6 °C in 5.5 Myr.

C2, early Visean (346.7-338.8 Ma). Conodont oxygen isotopes in South China [14] suggest a cooling event in the early Visean. The magnitude and duration of this cooling was 3.8 °C and 2.4 Myr.

C3, late Visean and Serpukhovian (338.8-323.2 Ma). Conodont oxygen isotopes from Europe [13] suggest that the largest magnitude of climate change in this time interval was a cooling event that happened in the Serpukhovian. Sea surface temperatures decreased ~3.8 °C in ~2.4 Myr.

C4, Bashkirian (323.2-315.2 Ma). Conodont oxygen isotopes in South China [14] suggest a slow cooling event in the early Bashkirian. The magnitude and duration of this cooling was 1.6 °C and 5.0 Myr.

C5, Moscovian and Kasimovian (315.2-303.7 Ma). Conodont oxygen isotope records from South China [14] suggest a short warming event in the early Moscovian with a magnitude of 3.4 °C in 1.7 Myr.

C6, Gzhelian (303.7-298.9 Ma). Conodont oxygen isotopes in South China [14] suggest a warming event in this interval with a magnitude of 1.0 °C in 2.9 Myr.

P1, Asselian and Sakmarian (298.9-290.1 Ma). Conodont oxygen isotope records from South China [14] suggest two cooling events in the Asselian and Sakmarian. The magnitude and duration of the larger of the two were 5.3 °C and 2.4 Myr.

P2, Artinskian (290.1-279.3 Ma). Conodont oxygen isotopes in South China [14] suggest a small warming event in the late Artinskian with a magnitude of 1.2 °C over 2.8 Myr.

P3, Kungurian and Roadian (279.3-268.8 Ma). Conodont oxygen isotope records from South China [14] suggest a minor warming in the late Kungurian-Roadian with a magnitude of 1.3 °C in 5.2 Myr.

Middle Permian Wordian and Capitanian

P4, Wordian and Capitanian (268.8-259.8 Ma). The temperature change during the Wordian and Capitanian (P4) are from high-resolution conodont oxygen isotope records in South China [21]. Ages derive from the Geological Time Scale 2012 [16] and U-Pb dates [22]. The Wordian and Capitanian interval was the last stage of the Late Paleozoic Ice Age, which would have contributed to the decrease of seawater $\delta^{18}O$ [20]. However, the most significant and rapid change of temperature in P4 was an increase in conodont $\delta^{18}O$ at the end-Capitanian, indicating a cooling event with a magnitude of 5.9 °C in ~1 Myr.

Late Permian

P5, Wuchiapingian and Changhsingian (259.8-251.9 Ma).

Temperature changes in the Late Permian are from conodont $\delta^{18}O$ in South China [23] with durations constrained with U-Pb dating [24]. We assumed an oxygen isotope value of seawater of -1‰ (VSMOW) for an ice-free world during the Late Permian [7] [8]. The most significant change of climate in the P5 interval was a rapid warming event at the end-Permian. Conodont oxygen isotopes measured with in situ secondary ion mass spectrometry (SIMS) at Meishan, Dajianggou, and

Liangfengya (South China) [23] suggest that sea surface temperature increased 11.1 °C, 8.5 °C, and 9.3 °C, respectively around the Permian-Triassic boundary. This temperature climb is consistent with the trend derived from conodont oxygen isotope measurements using the conventional isotope ratio mass spectrometry (IRMS) from South China, Iran, and Armenia [25-27]. The U-Pb dates of Burgess et al. suggest the duration of this warming event was >61 kyr (from 251.941 to 251.880 Ma) [24].

Early and Middle Triassic

T1, Induan-Anisian (251.9-247.2 Ma). The temperature change during the T1 interval derives from high-resolution conodont oxygen isotope records in South China[28]. Ages are from the Geological Time Scale 2012[29]. Conodont oxygen isotope data in South China suggest two significant temperature peaks in the Early Triassic. The largest warming happened in the Smithian with a magnitude of 6.1 °C in ~770 kyr[28].

Middle Triassic and Late Triassic

The magnitude and duration of temperature change of three bins (i.e., T2, Anisian and Ladinian; T3, Carnian; and T4, Norian) are from high-resolution conodont oxygen isotope records in Europe [30]. Oxygen isotope data from upwelling systems and cooler water conodont biofacies (e.g., *Gladigondolella* biofacies) were excluded from the reconstruction of sea surface temperature [30] (see Methods in main text). Temperature variations in the Rhaetian (T5) derive from oyster oxygen isotope records in the UK [31]. Only oxygen isotope values from well-preserved oyster (*Liostrea*) shells were selected for calculation of sea surface temperature. Ages derive from the Geological Time Scale 2012 [29]. We assumed -1‰ (VSMOW) as the oxygen isotope value of seawater for an ice-free world in the Triassic [7,8].

T2, Anisian and Ladinian (247.2-237 Ma). Conodont oxygen isotopes from Europe suggest a long-term cooling during the Ladinian [30]. Sea surface temperature decreased ~4.3 °C in ~3.9 Myr.

T3, Carnian (237-228.4 Ma). Conodont oxygen isotope data from South China suggest a significant warming during the late Carnian [32]. Conodont $\delta^{18}O$ from South China suggest that sea surface

temperatures increased ~3.5 °C in ~1.2 Myr. Climate warming has also been observed in the Carnian $\delta^{18}O$ record from European conodonts [30].

T4, Norian (228.4-209.5 Ma). Conodont oxygen isotopes from Europe show a temperature peak in the Norian [30]. Sea surface temperature climbed ~4 °C in the middle Norian before declining 4.5 °C in ~3.0 Myr. This temperature peak has also been observed in conodont data from the Canadian Cordillera [33].

T5, Rhaetian (209.5-201.3 Ma). Oxygen isotopes from well-preserved oysters (*Liostrea*) at Lavernock Point, UK suggest a significant warming event across the Triassic-Jurassic boundary interval [31]. Ages derive from the Geological Time Scale 2012 [34]. Sea surface temperature increased about 7.4 °C from the top of the Triassic Langport Member to the first occurrence of the ammonite *Psiloceras planorbis* [31].

Early Jurassic

The magnitude and duration of temperature change of three bins (i.e., J1, Hettangian and Sinemurian; J2, Pliensbachian; and J3, Toarcian) derive from belemnite and brachiopod oxygen isotope records in the UK, Spain and Portugal[35-39]. Ages derive from the Geological Time Scale 2012 [34]. We assumed -1‰ (VSMOW) as the oxygen isotope value of seawater for an ice-free world [7].

J1, Hettangian and Sinemurian (201.3-190.8 Ma). Belemnite oxygen isotopes from UK [37] suggest an abrupt decline of sea surface temperature in the middle Sinemurian, with a magnitude of 2.3 °C in ~1.9 Myr. This cooling trend has also been observed in the oyster $\delta^{18}O$ record [37].

J2, Pliensbachian (190.8-182.7 Ma). Brachiopod $\delta^{18}O$ from two locations in Portugal (Peniche and Tomar) show a consistent trend during the Pliensbachian and Toarcian [36]. Brachiopod oxygen isotopes from Portugal show a significant cooling event during the late Pliensbachian [36]. Sea surface temperatures decreased 5 °C in 2.5 Myr. This cooling event is also seen in belemnite $\delta^{18}O$ data from UK, Germany, and Portugal [40,41].

J3, Toarcian (182.7-174.1 Ma). Brachiopod oxygen isotopes from Portugal [36] suggest a significant warming event in the early Toarcian with a magnitude of ~5.4 °C in 1.4 Myr. This warming event is also seen in the $\delta^{18}O$ of belemnite from UK, Germany, Portugal, and Spain [38,40-43], and the $\delta^{18}O$ of fish teeth from Belgium, France, and Luxemburg [44]. $\delta^{18}O$ of belemnite from Reinosa, West Rodiles, La Almunia, and Tudanca of Spain [38,39] indicate a warming of 5.0 °C, 4.9 °C, 5.7 °C, and 6.5 °C, respectively.

Middle and Late Jurassic

Temperature change in the Middle Jurassic (J4) and Late Jurassic (J5) are from belemnite oxygen isotopes in the European realm representing shallow seas of the subtropical Tethyan area [45]. Temperature fluctuations in the Late Jurassic Kimmeridgian and Tithonian (J6) derive from $\delta^{18}O$ of Paris Basin oyster shells [46]. Ages are from the Geological Time Scale 2012 [34]. We assumed -1‰ (VSMOW) as the oxygen isotope value of seawater for an ice-free world [7].

J4, Middle Jurassic (Aalenian-Callovian, 174.1-163.5 Ma). Oxygen isotope data from Poland belemnites suggest a cooling in the Callovian [47]. Sea surface temperatures calculated from belemnite $\delta^{18}O$ decreased ~1.6 °C in 1.0 Myr.

J5, Oxfordian (163.5-157.3 Ma). Oxygen isotope data from Paris Basin oyster shells [46] suggest a rapaid warming during the late Oxfordian with a magnitude of 3.3 °C in ~0.7 Myr.

J6, Kimmeridgian and Tithonian (157.3-145 Ma). Oxygen isotope values from Paris Basin oyster shells suggest a significant warming event (~5.0°C) from the ammonoid *Pictonia baylei* Zone to the lower *Rasenia cymodoce* Zone (early Kimmeridgian) [46]. This warming trend is also seen in $\delta^{18}O$ records of belemnite rostra from Scotland [45] and brachiopods from India [48].

Cretaceous

The magnitudes of temperature change in two bins (K1 and K2) derive from belemnite oxygen isotope records, four bins (K3, K5, K6, and K8) utilize TEX_{86} (an organic paleothermometer) data, and three bins (K4, K7, and K8) use planktonic foraminiferal oxygen isotope values. Ages derive

from the Geological Time Scale 2012 [49]. We assumed -1‰ (VSMOW) as the oxygen isotope value of seawater for an ice-free world [7].

K1, Berriasian and Valanginian (145-133.9 Ma). Oxygen isotope data from Tethyan belemnites show many short-term swings during the Berriasian and Valanginian, which were probably caused by variations in polar ice-volume [50,51]. The long-term trend in the $\delta^{18}O$ record of belemnites during the Valanginian likely reflects temperature change since this trend is consistent with changes observed in Mg/Ca (reflecting calcification temperature) of belemnites [50]. Belemnite $\delta^{18}O$ suggest that sea surface temperature decreased ~2.4 °C during the middle Valanginian[50].

K2, Hauterivian and Barremian (133.9-126.3 Ma). No glacial records have been reported in the K2 interval. Therefore, we assumed -1‰ (VSMOW) as the oxygen isotope value of seawater [7]. Oxygen isotope data from French belemnites suggest a warming event during the end-Hauterivian to early Barremian [52]. Sea surface temperature increased 1.8 °C in ~1.1 Myr in the Tethyan realm (Fig. 5 in ref. [52]). Belemnite oxygen isotope data from the UK show that this warming event also occurred in the Boreal realm [53].

K3, Aptian (126.3-113 Ma). The TEX_{86} values of samples from Deep Sea Drilling Project (DSDP) Site 398 show a warming event during Oceanic Anoxic Event (OAE) 1a followed by a rapid cooling during the early Aptian [54]. The TEX_{86} data show that sea surface temperature decrease of 2.6 °C in ~0.9 Myr [54].

K4, Albian (113-100.5 Ma). Oxygen isotope values of planktonic foraminifera from Ocean Drilling Program (ODP) Site 1052 suggest several temperature peaks during the late Albian [55]. The largest magnitude of climate warming and its duration were 2.1 °C and ~0.9 Myr.

K5, Cenomanian (100.5-93.9 Ma). The TEX_{86} data from the tropical Atlantic Ocean (ODP Sites 1258 and 1259) suggest a significant warming event during the Cenomanian [56]. The TEX_{86} data suggest that sea surface temperature increased 3.8 °C in ~3.3 Myr.

K6, Turonian-Santonian (93.9-83.6 Ma). The TEX_{86} data from ODP Site 1259 suggest a significant cooling event from the late Turonian to Santonian [56]. Sea surface temperature decreased 1.9 °C in ~5.4 Myr. This cooling trend is also seen in planktonic foraminifera $\delta^{18}O$ at ODP Site 1259 [57]. The short-term positive excursion of $\delta^{18}O$ in both the surface and deep ocean suggests a 200 kyr period of glaciation during the middle Turonian [57].

K7, Campanian (83.6-72.1 Ma). Oxygen isotope data of planktonic foraminifera from the eastern Indian Ocean (ODP Site 762C) show a significant cooling event during the early and middle Campanian[58]. Planktonic foraminifera (*Contusotruncana fornicate*) living in the surface mixed layer suggest that sea surface temperature decreased 4.9 °C in ~3.6 Myr. This cooling trend is also seen in planktonic foraminifera $\delta^{18}O$ data at ODP Site 1210B (northwestern Pacific Ocean)[58].

K8, Maastrichtian (72.1-66 Ma). The TEX_{86} data from Texas and New Jersey (USA) suggest a rapid cooling around the Cretaceous-Paleogene (K-Pg) boundary, coinciding with an iridium spike associated with the well-known bolide impact [59,60]. The TEX_{86} data from Texas and New Jersey[59,60] and oxygen isotope data of planktonic foraminifera from the Ancora and Bass River boreholes[61] suggest sea surface temperature declined 5.2 °C from the upper part of the terminal Cretaceous calcareous nannofossil Zone of *Micula prinsii* to the iridium spike layer. The U-Pb dates of Schoene et al. suggest the duration of this cooling event was ~121 kyr [62]. This cooling event has also been observed in the fossil plants from North Dakota, USA [63], suggesting that this was a global climate trend.

Paleogene and Neogene

Ages for time bins in the Paleogene and Neogene are from the Geological Time Scale 2012 [64,65].

Pg1, Paleocene (66-56 Ma). TEX_{86} data from the equatorial Atlantic suggest a rapid warming event around the Paleocene-Eocene boundary [66], known as the Paleocene-Eocene Thermal Maximum (PETM, 56 Ma). The TEX_{86} data from ODP Site 959 [66] and Nigeria [67] suggest that sea surface temperature increased ~5.1 °C in ~95 kyr. This warming has also been observed in the TEX_{86} records

at Bass River and Wilson Lake along the New Jersey Shelf [68] and in the planktonic foraminiferal $\delta^{18}O$ record in the Southern Ocean (ODP Site 690) [69].

Pg2, Ypresian (Eocene, 56-47.8 Ma). TEX_{86} data from the eastern equatorial Atlantic Ocean (ODP Site 959) show a warming event during the early Eocene, known as the Early Eocene Climatic Optimum[70]. This warming trend has also been observed in the Mg/Ca values of planktonic foraminifer *Morozovella* from western equatorial Pacific Ocean (ODP Site 865) [71]. TEX_{86} data from ODP Site 959[70] and South Dover Bridge [72] suggest tropical sea surface temperature increase 1.0 °C in ~3.3 Myr. This warming trend has also been observed in the surface ocean at high latitudes [72,73] and the global deep ocean [70].

Pg3, Lutetian (Eocene, 47.8-41.2 Ma). TEX_{86} data from the eastern equatorial Atlantic Ocean (ODP Site 959) show a long-term cooling during the Lutetian [70]. This cooling trend has also been observed in the Mg/Ca values of planktonic foraminifer *Morozovella* from western equatorial Pacific Ocean (ODP Site 865) [71]. TEX_{86} data from ODP Site 959[70] and South Dover Bridge [72] suggest that tropical sea surface temperature decreased ~1.7 °C decrease in ~1.2 Myr. TEX_{86} data from the Antarctic margin also show a cooling trend in this interval[73].

Pg4, Bartonian and Priabonian (Eocene, 41.2-33.9 Ma). Multiproxy climate records show a rapid cooling during the Eocene-Oligocene transition [70,74,75]. $\delta^{18}O$, and TEX_{86} data from low latitude regions [70,74,75] suggest sea surface temperature decreased ~2.4 °C in ~526 kyr. Multiproxy climate records from mid- and high-latitude oceans also show a similar cooling trend during the Eocene-Oligocene transition, suggesting a global trend [76,77].

Pg5, Oligocene (33.9-23.03 Ma). TEX_{86} data from DSDP 516F suggest that sea surface temperature decreased ~1.5 °C in ~2 Myr [78]. This cooling event has also been observed in alkenone and TEX_{86} temperature records from the mid-latitude Atlantic Ocean (IODP Sites U1404 and 1406A) [77,79].

Ng1, Aquitanian and Burdigalian (Early Miocene, 23.03-15.97 Ma). TEX_{86} data from the North Atlantic Ocean (DSDP Site 608) show a rapid cooling at the end of the Early Miocene [80,81]. Sea

surface temperature decreased ~2 °C in ~0.9 Myr. This short-term cooling event has also been observed in a carbonate clumped isotope (Δ47) record from Central European paleosols (Molasse Basin, Switzerland) [82].

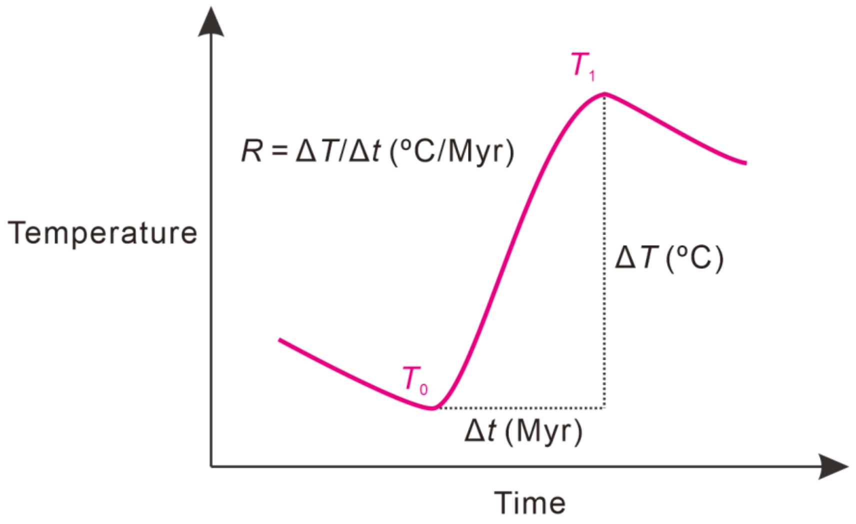

Supplementary Fig. 1 Schematic diagram illustrating calculation of the magnitude (ΔT) and rate (R) of temperature change for a given time interval (Δt). Myr, million years.

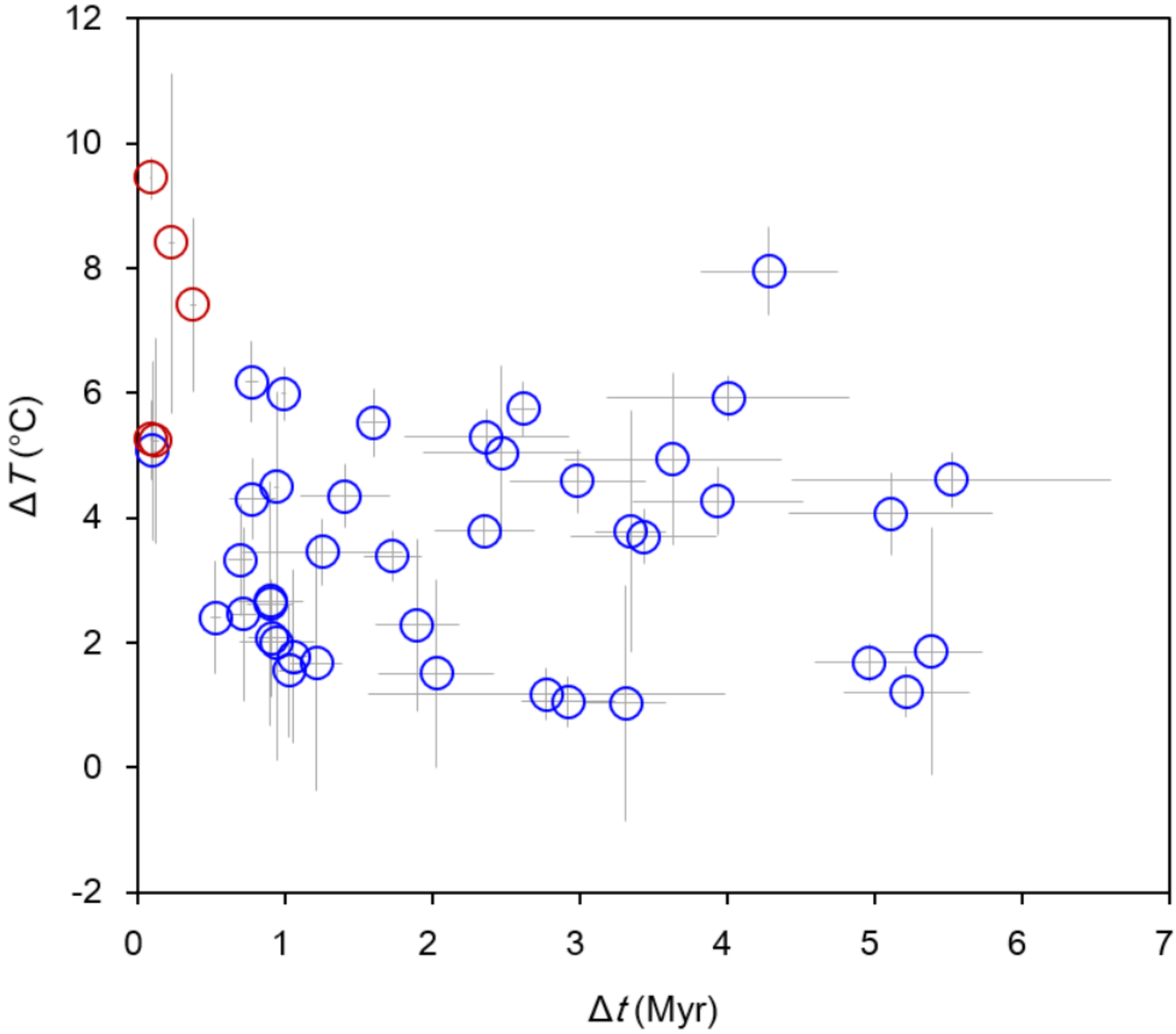

Supplementary Fig. 2 The relationship between timespan (Δ*t*) and magnitude (Δ*T*) of climate change over the past 450 million years. There is no correlation between timespan and magnitude (n = 45, $\rho = -0.148$, $P = 0.331$). Red dots represent the Big Five extinctions. Horizontal and vertical bars represent mean ± 1 x standard deviation. n = the sample size used to derive statistics.

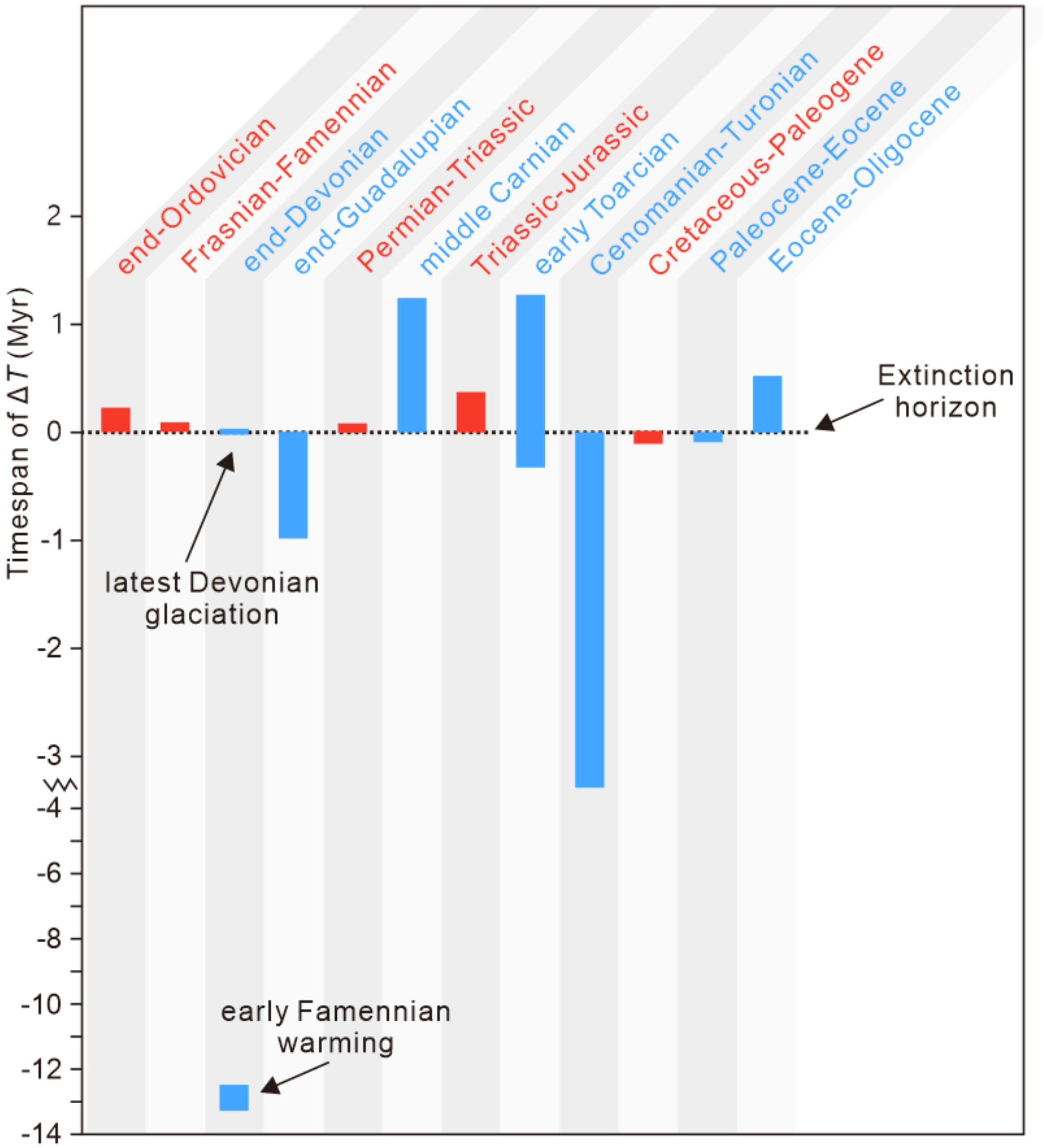

Supplementary Fig. 3 The relative timing of temperature change (Δ*T*) and extinction for the major (red) and minor (blue) extinctions during the past 450 million years. The dashed line is the relative position of the extinction horizon in relation to the timespan of the largest magnitude climate changes (colored bars) within the time interval containing the extinction. For the list of major and minor extinctions, ages and ΔT see Supplementary Table 3. Red and blue bars represent Big Five and minor mass extinctions, respectively.

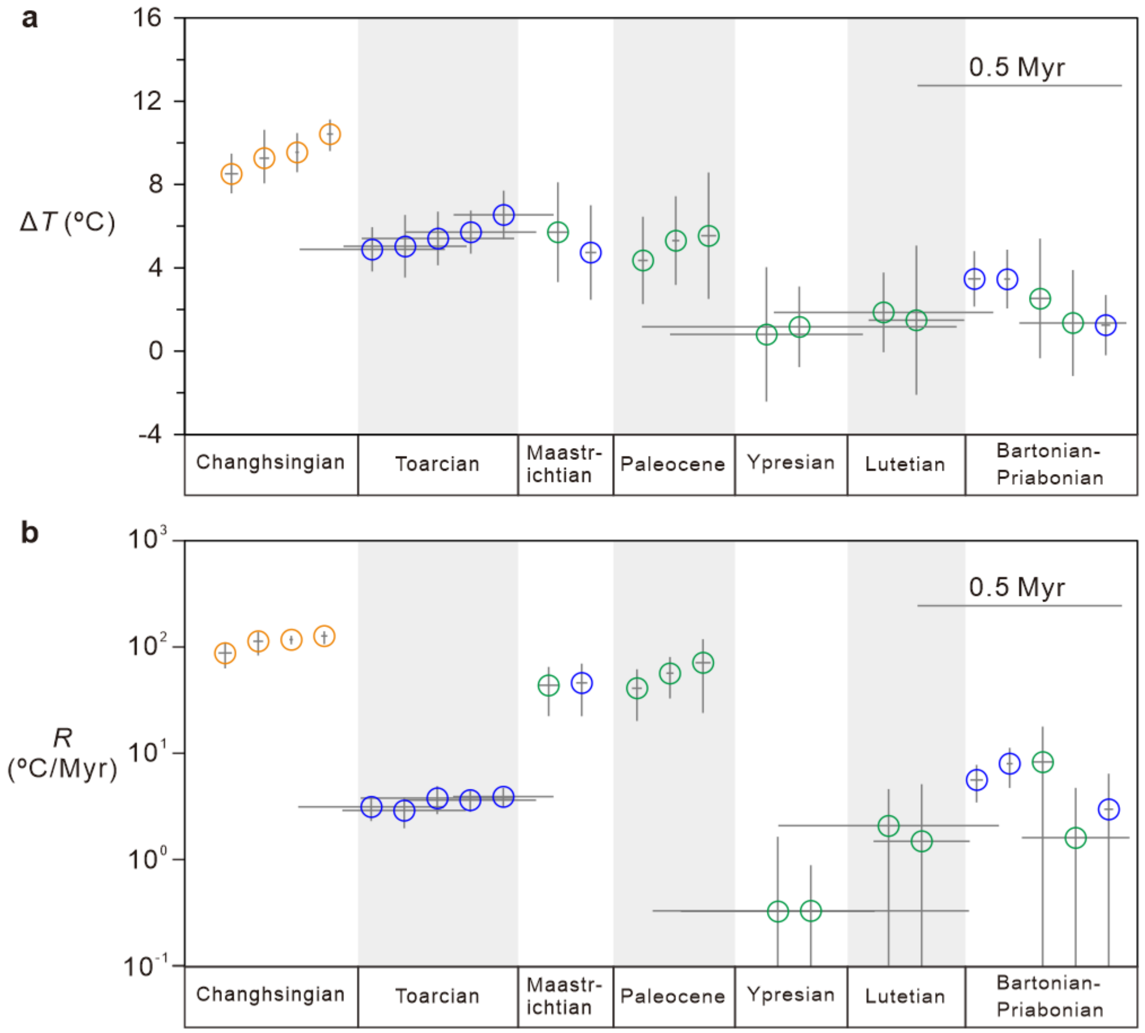

Supplementary Fig. 4 Comparisons of the magnitude (Δ*T*) and rate (*R*) of temperature change between different temperature-proxies or between different sites for seven time intervals. a, The magnitude of temperature change. **b,** The rate of temperature change. Orange, blue, and green circles represent conodont $\delta^{18}O$, carbonate fossil $\delta^{18}O$, and TEX_{86}, respectively. $\delta^{18}O_{conodont}$ data in the Changhsingian are from Dajianggou, Liangfengya, Meishan of South China using in situ SIMS technique [23] and Armenia using bulk conodont apatite [27]. $\delta^{18}O_{belemnite}$ and $\delta^{18}O_{brachiopod}$ data in the Toarcian are from Spain [38,83] and Portugal [36], respectively. TEX_{86} and $\delta^{18}O_{foraminifer}$ data in the Maastrichtian are from New Jersey [59,60] and Ancora-Bass River [61], respectively. TEX_{86} data in the Paleocene are from ODP Site 959 [66] and Nigeria [67]. TEX_{86} data in the Ypresian are from ODP Site 959 [70] and the South Dover Bridge Core, Maryland [72]. TEX_{86} data in the Bartonian-Priabonian are from ODP Site 959 [70] and St. Stephens Quarry, Alabama [74]. $\delta^{18}O_{foraminifer}$ data in the Bartonian-Priabonian are Gulf of Mexico [74] and Tanzania [75]. Horizontal and vertical bars show mean ± 1 x standard deviation.

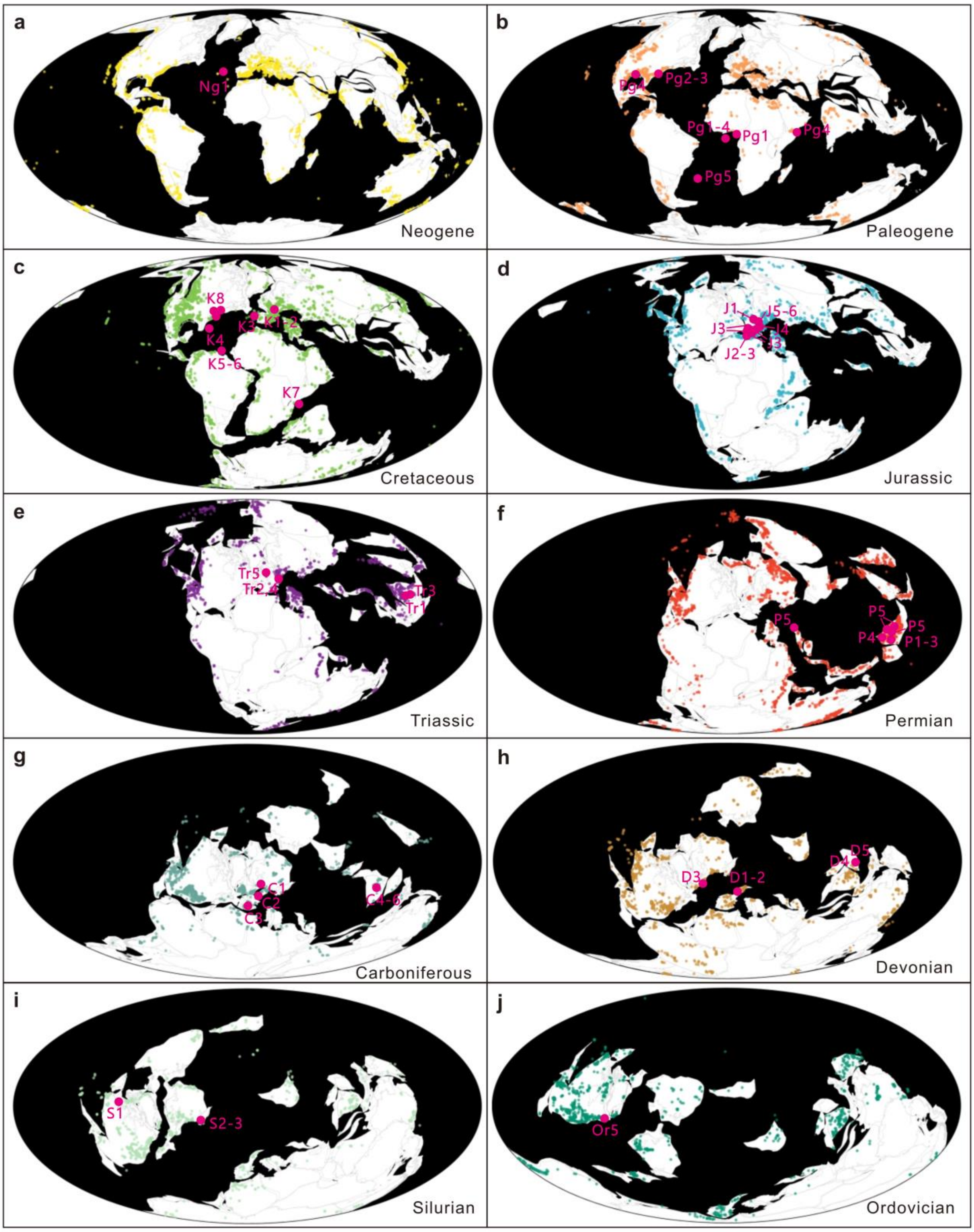

Supplementary Fig. 5 Locations of paleobiological data (small dots) and paleo-temperature data (big dots). a, Neogene; **b,** Paleogene; **c,** Cretaceous; **d,** Jurassic; **e,** Triassic; **f,** Permian; **g,** Carboniferous; **h,** Devonian; **i,** Silurian; **j,** Ordovician. Paleogeographic map and fossil data are from Paleobiology Database (PBDB, http://paleobiodb.org).

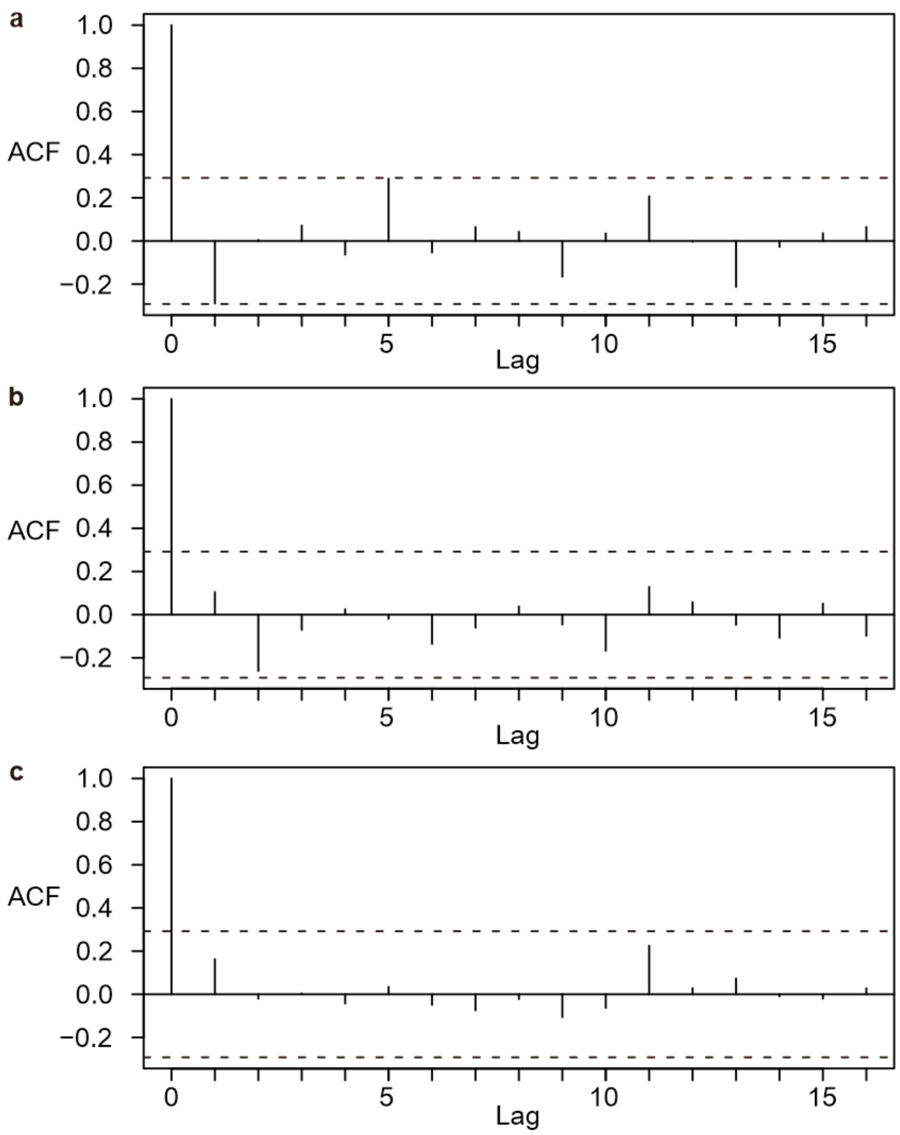

Supplementary Fig. 6 Results of autocorrelation function (ACF) for Δ*T* (a), log *R* (b), and GF extinction (c). Dotted lines represent 95% confidence intervals.

Supplementary Table 1 Results of correlation analysis among extinction rates of marine animals and climate change.

Type	Variables	n	Spearman's ρ	*P*-value
All bins	GF-PBDB vs *ΔT*	45	0.629	3.64E-06
	GF-PBDB vs *R*	45	0.567	4.92E-05
	3T-PBDB vs *ΔT*	45	0.593	1.78E-05
	3T-PBDB vs *R*	45	0.539	1.32E-04
Excluding the Big Five extinctions	GF-PBDB vs *ΔT*	40	0.536	3.68E-04
	GF-PBDB vs *R*	40	0.422	6.72E-03
	3T-PBDB vs *ΔT*	40	0.473	2.05E-03
	3T-PBDB vs *R*	40	0.364	2.11E-02
Cooling bins	GF-PBDB vs *ΔT*	25	-0.533	6.07E-03
	GF-PBDB vs *R*	25	-0.308	1.34E-01
	3T-PBDB vs *ΔT*	25	-0.512	8.96E-03
	3T-PBDB vs *R*	25	-0.335	1.02E-01
Warming bins	GF-PBDB vs *ΔT*	20	0.756	1.14E-04
	GF-PBDB vs *R*	20	0.782	4.65E-05
	3T-PBDB vs *ΔT*	20	0.809	1.57E-05
	3T-PBDB vs *R*	20	0.723	3.13E-04
All bins	GFS-PBDB vs *ΔT*	45	0.610	8.60E-06
	GFS-PBDB vs *R*	45	0.571	4.28E-05
	3TS-PBDB vs *ΔT*	45	0.640	2.21E-06
	3TS-PBDB vs *R*	45	0.505	3.99E-04
All bins	Paleolatitude vs *ΔT*	45	-0.138	0.367
	Paleolatitude vs *R*	45	-0.005	0.974

Δ*T*, the magnitude of temperature change; *R*, the of temperature change; GF, gap-filler rate of extinction; 3T, three-timer rate of extinction; GFS, gap-filler rate of shallow-water animal extinction; 3T, three-timer rate of shallow-water animal extinction; PBDB, Paleobiology Database. The statistical test was two-sided and no adjustments were made for multiple comparisons.

Supplementary Table 2 Results of Pearson-Filon test using R-package cocor. The results suggest that there are no significant differences between correlation coefficients of GF extinction rate and $\Delta T/R$ under warming and cooling events. There are no significant differences between correlation coefficients of GF extinction rate and $\Delta T/R$ from Paleozoic, Mesozoic, and Cenozoic.

Variables	Pearson and Filon's *z*	*P* value
GF extinction rates ~ ΔT | GF extinction rates ~ logR	0.34816	0.7277
GF extinction rates ~ warming ΔT | GF extinction rates ~ cooling ΔT	1.5757	0.1151
GF extinction rates ~ warming logR | GF extinction rates ~ cooling logR	1.2252	0.2205
Cenozoic extinction rates ~ Cenozoic ΔT | Mesozoic extinction rates ~ Mesozoic ΔT	0.42722	0.6692
Cenozoic extinction rates ~ Cenozoic ΔT | Paleozoic extinction rates ~ Paleozoic ΔT	0.15812	0.8744
Mesozoic extinction rates ~ Mesozoic ΔT | Paleozoic extinction rates ~ Paleozoic ΔT	0.82373	0.4101
Cenozoic extinction rates ~ Cenozoic logR | Mesozoic extinction rates ~ Mesozoic logR	0.53307	0.594
Cenozoic extinction rates ~ Cenozoic logR | Paleozoic extinction rates ~ Paleozoic logR	0.44584	0.6557
Mesozoic extinction rates ~ Mesozoic logR | Paleozoic extinction rates ~ Paleozoic logR	0.06187	0.9507

Supplementary Table 3 The relative timing of temperature change (Δ*T*) and extinction for the major and minor extinctions during the past 450 million years.

Bin name	Major/minor extinction	Taxa that suffered significant extinction	Onset age of extinction (Myr)	t_0 (Ma)	t_1 (Ma)	Δt (Myr)	References for extinction event
Pg4	Eocene-Oligocene extinction	bivalve, plant, gastropod, planktonic foraminifer	34	34	33.45	0.55	Retallack et al., 2004; Pearson et al., 2008; Vandenberghe et al., 2012
Pg1	Paleocene-Eocene extinction	benthic foraminifer, ostracod, phytoplankton	56	56.05	56	0.05	Hallam and Wignall, 1997; Thomas and Shackleton, 1996; Gibbs et al., 2006; Vandenberghe et al., 2012
K8	K-Pg extinction	ammonoid, belemnite, benthic foraminifer, bivalve, brachiopod, bryozoan, dinosaur, lizard, marine reptile, planktonic foraminifer, phytoplankton, plant, snake	66.016	66.19	66.02	0.174	Hallam and Wignall, 1997; Longrich et al., 2011; 2012; Schoene et al., 2019
K5	Cenomanian-Turonian extinction	benthic foraminifer, planktonic foraminifer, molluscan, phytoplankton	94.4	97.69	94.4	3.29	Hallam and Wignall, 1997; Wan et al., 2013; Parente et al., 2008; Ogg and Hinnov, 2012
J3	early Toarcian extinction	bivalve, brachiopod, large foraminifer	182.7	182.94	181.5	1.43	Hallam and Wignall, 1997; Caswell et al., 2009; Ogg et al., 2012; Jiang et al., 2020
Tr3	middle Carnian extinction	ammonoid, bivalve, brachiopod, coral, conodont, foraminifer, gastropod, ostracod, radiolarian, sponge	233.1	233.1	231.9	1.2	Ogg et al., 2012; Dal Corso et al., 2020
Tr5	Triassic-Jurassic extinction	ammonoid, bivalve, brachiopod, coral, conodont, foraminifer, nanofossil, ostracod, plant, radiolaria, sponge, Tabulozoan	201.5	201.5	201.1	0.4	Hallam and Wignall, 1997; Ogg et al., 2012; Wignall and Atkinson, 2020
P5	Permian-Triassic extinction	ammonoid, bivalve, brachiopod, conodont, calcareous algae, coral, fusulinid, gastropod, ostracod, plant, radiolaria, sponge, tetrapod, trilobite	251.94	251.94	251.9	0.061	Shen et al., 2011; Song et al., 2013; Benton and Newell, 2014; Burgess et al., 2014
P4	end-Guadalupian extinction	ammonoid, bivalve, brachiopod, bryozoan, coral, fusulinid, plant	259.1	259.8	259.1	0.7	Stanley and Yang, 1994; Hallam and Wignall, 1997; Brayard et al., 2009; Bond and Wignall, 2010; Zhong et al., 2014
D5	end-Devonian extinction	ammonoid, conodont, marine and terrestrial vertebrates, stromatoporoid, trilobite	358.97	372.19	371.4	0.78	Hallam and Wignall, 1997; Kaiser et al., 2006; Sallan and Coates, 2010; Myrow et al., 2014
D4	Frasnian-Famennian extinction	ammonoid, brachiopod, conodont, coral, fish, stromatoporoid, trilobite, ostracod	372.3	372.29	372.2	0.08	Hallam and Wignall, 1997; Becker et al., 2012; Huang et al., 2018
Or5	end-Ordovician extinction	acritarch, brachiopod, bryozoan, conodont, coral, graptolite, sponge, stromatoporoid, trilobite	443.14	443.14	442.9	0.22	Hallam and Wignall, 1997; Ling et al., 2019; Rong et al., 2020

For the detailed temperature data see Source Data. Data of extinctions are from references [2,10,11,22,24,29,34,49,62,64,75,84-105]. Data of temperature change are from Source Data. Red and blue colours represent Big Five and minor mass extinctions, respectively.

References

1 Finnegan, S. *et al.* The magnitude and duration of Late Ordovician–Early Silurian glaciation. *Science* **331**, 903–906 (2011).

2 Ling, M.-X. *et al.* An extremely brief end Ordovician mass extinction linked to abrupt onset of glaciation. *Solid Earth Sci.* **4**, 190-198 (2019).

3 Trotter, J. A., Williams, I. S., Barnes, C. R., Lecuyer, C. & Nicoll, R. S. Did cooling oceans trigger Ordovician biodiversification? Evidence from conodont thermometry. *Science* **321**, 550–554 (2008).

4 Achab, A., Asselin, E., Desrochers, A., Riva, J. & Farley, C. Chitinozoan biostratigraphy of a new Upper Ordovician stratigraphic framework for Anticosti Island, Canada. *Geol. Soc. Am. Bull.* **123**, 186-205 (2011).

5 Trotter, J. A., Williams, I. S., Barnes, C. R., Männik, P. & Simpson, A. New conodont $\delta^{18}O$ records of Silurian climate change: Implications for environmental and biological events. *Palaeogeogr., Palaeoclimatol., Palaeoecol.* **443**, 34-48 (2016).

6 Melchin, M. J. *et al.* in *The Geologic Time Scale* (eds Felix M. Gradstein, James G. Ogg, Mark D. Schmitz, & Gabi M. Ogg) 525-558 (Elsevier, 2012).

7 Savin, S. M. The History of the Earth's surface temperature during the past 100 million years. *Annu. Rev. Earth Planet Sci.* **5**, 319-355 (1977).

8 Crowley, J. K. & Berner, R. A. CO_2 and climate change. *Science* **292**, 870–872 (2001).

9 Joachimski, M. M. *et al.* Devonian climate and reef evolution: Insights from oxygen isotopes in apatite. *Earth Planet. Sci. Lett.* **284**, 599-609 (2009).

10 Becker, R. T., Gradstein, F. M. & Hammer, O. in *The Geologic Time Scale* (eds Felix M. Gradstein, James G. Ogg, Mark D. Schmitz, & Gabi M. Ogg) 559-601 (Elsevier, 2012).

11 Huang, C., Joachimski, M. M. & Gong, Y. Did climate changes trigger the Late Devonian Kellwasser Crisis? Evidence from a high-resolution conodont $\delta^{18}O_{PO4}$ record from South China. *Earth Planet. Sci. Lett.* **495**, 174-184 (2018).

12 Caputo, M. V., de Melo, J. G., Streel, M. & Isbell, J. L. Late Devonian and early Carboniferous glacial records of South America. *Geol. Soc. Am. Spec. Pap.* **441**, 161-173 (2008).

13 Buggisch, W., Joachimski, M. M., Sevastopulo, G. & Morrow, J. R. Mississippian $\delta^{13}C_{carb}$ and conodont apatite $\delta^{18}O$ records — Their relation to the Late Palaeozoic Glaciation. *Palaeogeogr., Palaeoclimatol., Palaeoecol.* **268**, 273-292 (2008).

14 Chen, B. *et al.* Ice volume and paleoclimate history of the Late Paleozoic Ice Age from conodont apatite oxygen isotopes from Naqing (Guizhou, China). *Palaeogeogr., Palaeoclimatol., Palaeoecol.* **448**, 151-161 (2016).

15 Davydov, V. I., Korn, D., Schmitz, M. D., Gradstein, F. M. & Hammer, O. in *The Geologic Time Scale* (eds Felix M. Gradstein, James G. Ogg, Mark D. Schmitz, & Gabi M. Ogg) 603-651 (Elsevier, 2012).

16 Henderson, C. M., Davydov and, V. I., Wardlaw, B. R., Gradstein, F. M. & Hammer, O. in *The Geologic Time Scale* (eds Felix M. Gradstein, James G. Ogg, Mark D. Schmitz, & Gabi M. Ogg) 653-679 (Elsevier, 2012).

17 Crowell, J. C. *Pre-Mesozoic Ice Ages: Their Bearing on Understanding the Climate System*. Vol. 192 (Geological Society of America, 1999).

18 Schrag, D. P., Hampt, G. & Murray, D. W. Pore fluid constraints on the temperature and oxygen isotopic composition of the glacial ocean. *Science* **272**, 1930-1932 (1996).

19 González-Bonorino, G. & Eyles, N. Inverse relation between ice extent and the late Paleozoic glacial record of Gondwana. *Geology (Boulder)* **23**, 1015-1018 (1995).

20 Montañez, I. P. & Poulsen, C. J. The Late Paleozoic Ice Age: An evolving paradigm. *Annu. Rev. Earth Planet Sci.* **41**, 629-656 (2013).

21 Chen, B. *et al.* Permian ice volume and palaeoclimate history: Oxygen isotope proxies revisited. *Gondwana Res.*

24, 77-89 (2013).
22 Zhong, Y.-T., He, B., Mundil, R. & Xu, Y.-G. CA-TIMS zircon U–Pb dating of felsic ignimbrite from the Binchuan section: Implications for the termination age of Emeishan large igneous province. *Lithos* **204**, 14-19 (2014).
23 Chen, J. *et al.* High-resolution SIMS oxygen isotope analysis on conodont apatite from South China and implications for the end-Permian mass extinction. *Palaeogeogr., Palaeoclimatol., Palaeoecol.* **448**, 26-38 (2016).
24 Burgess, S. D., Bowring, S. & Shen, S.-z. High-precision timeline for Earth's most severe extinction. *Proc. Natl. Acad. Sci. USA* **111**, 3316-3321 (2014).
25 Joachimski, M. M. *et al.* Climate warming in the latest Permian and the Permian–Triassic mass extinction. *Geology* **40**, 195-198 (2012).
26 Schobben, M., Joachimski, M. M., Korn, D., Leda, L. & Korte, C. Palaeotethys seawater temperature rise and an intensified hydrological cycle following the end-Permian mass extinction. *Gondwana Res.* **26**, 675-683 (2014).
27 Joachimski, M. M., Alekseev, A. S., Grigoryan, A. & Gatovsky, Y. A. Siberian Trap volcanism, global warming and the Permian-Triassic mass extinction: New insights from Armenian Permian-Triassic sections. *Geol. Soc. Am. Bull.* **132**, 427-443 (2020).
28 Sun, Y. *et al.* Lethally hot temperatures during the Early Triassic greenhouse. *Science* **338**, 366-370 (2012).
29 Ogg, J. G. in *The Geologic Time Scale* (eds Felix M. Gradstein, James G. Ogg, Mark D. Schmitz, & Gabi M. Ogg) 681-730 (Elsevier, 2012).
30 Trotter, J. A., Williams, I. S., Nicora, A., Mazza, M. & Rigo, M. Long-term cycles of Triassic climate change: a new δ^{18}O record from conodont apatite. *Earth Planet. Sci. Lett.* **415**, 165-174 (2015).
31 Korte, C., Hesselbo, S. P., Jenkyns, H. C., Rickaby, R. E. & Spötl, C. Palaeoenvironmental significance of carbon-and oxygen-isotope stratigraphy of marine Triassic–Jurassic boundary sections in SW Britain. *J. Geol. Soc. Lond.* **166**, 431-445 (2009).
32 Sun, Y. D. *et al.* Climate warming, euxinia and carbon isotope perturbations during the Carnian (Triassic) Crisis in South China. *Earth Planet. Sci. Lett.* **444**, 88-100 (2016).
33 Sun, Y. D., Orchard, M. J., Kocsis, Á. T. & Joachimski, M. M. Carnian–Norian (Late Triassic) climate change: Evidence from conodont oxygen isotope thermometry with implications for reef development and Wrangellian tectonics. *Earth Planet. Sci. Lett.* **534**, 116082 (2020).
34 Ogg, J. G., Hinnov, L. A. & Huang, C. in *The Geologic Time Scale* (eds Felix M. Gradstein, James G. Ogg, Mark D. Schmitz, & Gabi M. Ogg) 731-791 (Elsevier, 2012).
35 Suan, G. *et al.* Duration of the Early Toarcian carbon isotope excursion deduced from spectral analysis: Consequence for its possible causes. *Earth Planet. Sci. Lett.* **267**, 666-679 (2008).
36 Suan, G. *et al.* Secular environmental precursors to Early Toarcian (Jurassic) extreme climate changes. *Earth Planet. Sci. Lett.* **290**, 448-458 (2010).
37 Korte, C. & Hesselbo, S. P. Shallow marine carbon and oxygen isotope and elemental records indicate icehouse-greenhouse cycles during the Early Jurassic. *Paleoceanography* **26**, PA4219 (2011).
38 Rosales, I., Quesada, S. & Robles, S. Paleotemperature variations of Early Jurassic seawater recorded in geochemical trends of belemnites from the Basque–Cantabrian basin, northern Spain. *Palaeogeogr., Palaeoclimatol., Palaeoecol.* **203**, 253-275 (2004).
39 Gómez, J. J. & Goy, A. Warming-driven mass extinction in the Early Toarcian (Early Jurassic) of northern and central Spain. Correlation with other time-equivalent European sections. *Palaeogeogr., Palaeoclimatol., Palaeoecol.* **306**, 176-195 (2011).
40 Jenkyns, H. C., Jones, C. E., Gröcke, D. R., Hesselbo, S. P. & Parkinson, D. N. Chemostratigraphy of the Jurassic System: applications, limitations and implications for palaeoceanography. *J. Geol. Soc. Lond.* **159**, 351-378 (2002).
41 Korte, C. *et al.* Jurassic climate mode governed by ocean gateway. *Nat. Comm.* **6**, 10015 (2015).

42 McArthur, J. M., Donovan, D. T., Thirlwall, M. F., Fouke, B. W. & Mattey, D. Strontium isotope profile of the early Toarcian (Jurassic) oceanic anoxic event, the duration of ammonite biozones, and belemnite palaeotemperatures. *Earth Planet. Sci. Lett.* **179**, 269-285 (2000).

43 Gómez, J. J., Goy, A. & Canales, M. L. Seawater temperature and carbon isotope variations in belemnites linked to mass extinction during the Toarcian (Early Jurassic) in Central and Northern Spain. Comparison with other European sections. *Palaeogeogr Palaeoclimatol Palaeoecol* **258**, 28-58 (2008).

44 Dera, G. *et al.* Water mass exchange and variations in seawater temperature in the NW Tethys during the Early Jurassic: Evidence from neodymium and oxygen isotopes of fish teeth and belemnites. *Earth Planet. Sci. Lett.* **286**, 198-207 (2009).

45 Dera, G. *et al.* Climatic ups and downs in a disturbed Jurassic world. *Geology* **39**, 215–218 (2011).

46 Brigaud, B., Pucéat, E., Pellenard, P., Vincent, B. & Joachimski, M. M. Climatic fluctuations and seasonality during the Late Jurassic (Oxfordian–Early Kimmeridgian) inferred from $\delta^{18}O$ of Paris Basin oyster shells. *Earth Planet. Sci. Lett.* **273**, 58-67 (2008).

47 Wierzbowski, H., Dembicz, K. & Praszkier, T. Oxygen and carbon isotope composition of Callovian–Lower Oxfordian (Middle–Upper Jurassic) belemnite rostra from central Poland: a record of a Late Callovian global sea-level rise? *Palaeogeogr., Palaeoclimatol., Palaeoecol.* **283**, 182-194 (2009).

48 Alberti, M., Fürsich, F. T. & Pandey, D. K. The Oxfordian stable isotope record ($\delta^{18}O$, $\delta^{13}C$) of belemnites, brachiopods, and oysters from the Kachchh Basin (western India) and its potential for palaeoecologic, palaeoclimatic, and palaeogeographic reconstructions. *Palaeogeogr., Palaeoclimatol., Palaeoecol.* **344-345**, 49-68 (2012).

49 Ogg, J. G., Hinnov, L. A. & Huang, C. in *The Geologic Time Scale* (eds Felix M. Gradstein, James G. Ogg, Mark D. Schmitz, & Gabi M. Ogg) 793-853 (Elsevier, 2012).

50 McArthur, J. M. *et al.* Palaeotemperatures, polar ice-volume, and isotope stratigraphy (Mg/Ca, $\delta^{18}O$, $\delta^{13}C$, $^{87}Sr/^{86}Sr$): The Early Cretaceous (Berriasian, Valanginian, Hauterivian). *Palaeogeogr., Palaeoclimatol., Palaeoecol.* **248**, 391-430 (2007).

51 Alley, N. F. & Frakes, L. A. First known Cretaceous glaciation: Livingston Tillite Member of the Cadna - owie Formation, South Australia. *Aust. J. Earth Sci.* **50**, 139-144 (2003).

52 Bodin, S. *et al.* Early Cretaceous (late Berriasian to early Aptian) palaeoceanographic change along the northwestern Tethyan margin (Vocontian Trough, southeastern France): $\delta^{13}C$, $\delta^{18}O$ and Sr-isotope belemnite and whole-rock records. *Cretaceous Res.* **30**, 1247-1262 (2009).

53 McArthur, J. M. *et al.* Belemnites of Valanginian, Hauterivian and Barremian age: Sr-isotope stratigraphy, composition ($^{87}Sr/^{86}Sr$, $\delta^{13}C$, $\delta^{18}O$, Na, Sr, Mg), and palaeo-oceanography. *Palaeogeogr., Palaeoclimatol., Palaeoecol.* **202**, 253-272 (2004).

54 Naafs, B. D. A. & Pancost, R. D. Sea-surface temperature evolution across Aptian Oceanic Anoxic Event 1a. *Geology* **44**, 959-962 (2016).

55 Wilson, P. A. & Norris, R. D. Warm tropical ocean surface and global anoxia during the mid-Cretaceous period. *Nature* **412**, 425-429 (2001).

56 Forster, A., Schouten, S., Baas, M. & Damsté, J. S. S. Mid-Cretaceous (Albian–Santonian) sea surface temperature record of the tropical Atlantic Ocean. *Geology* **35**, 919-922 (2007).

57 Bornemann, A. *et al.* Isotopic evidence for glaciation during the Cretaceous supergreenhouse. *Science* **319**, 189-192 (2008).

58 Falzoni, F., Petrizzo, M. R., MacLeod, K. G. & Huber, B. T. Santonian–Campanian planktonic foraminifera from Tanzania, Shatsky Rise and Exmouth Plateau: Species depth ecology and paleoceanographic inferences. *Mar. Micropaleontol.* **103**, 15-29 (2013).

59 Vellekoop, J. *et al.* Rapid short-term cooling following the Chicxulub impact at the Cretaceous–Paleogene boundary. *Proc. Natl. Acad. Sci. USA* **111**, 7537-7541 (2014).
60 Vellekoop, J. *et al.* Evidence for Cretaceous-Paleogene boundary bolide “impact winter” conditions from New Jersey, USA. *Geology* **44**, 619-622 (2016).
61 Olsson, R. K., Wright, J. D. & Miller, K. G. Paleobiogeography of *Pseudotextularia elegans* during the latest Maastrichtian global warming event. *J. Foram. Res.* **31**, 275-282 (2001).
62 Schoene, B. *et al.* U-Pb constraints on pulsed eruption of the Deccan Traps across the end-Cretaceous mass extinction. *Science* **363**, 862-866 (2019).
63 Wilf, P., Johnson, K. R. & Huber, B. T. Correlated terrestrial and marine evidence for global climate changes before mass extinction at the Cretaceous-Paleogene boundary. *Proc. Natl. Acad. Sci. USA* **100**, 599-604 (2003).
64 Vandenberghe, N. *et al.* in *The Geologic Time Scale* (eds Felix M. Gradstein, James G. Ogg, Mark D. Schmitz, & Gabi M. Ogg) 855-921 (Elsevier, 2012).
65 Hilgen, F. J. *et al.* in *The Geologic Time Scale* (eds Felix M. Gradstein, James G. Ogg, Mark D. Schmitz, & Gabi M. Ogg) 923-978 (Elsevier, 2012).
66 Frieling, J. *et al.* Widespread warming before and elevated barium burial during the Paleocene-Eocene Thermal Maximum: evidence for methane hydrate release? *Paleoceanogr. Paleoclimatol.* **34**, 546-566 (2019).
67 Frieling, J. *et al.* Extreme warmth and heat-stressed plankton in the tropics during the Paleocene-Eocene Thermal Maximum. *Sci. Adv.* **3**, e1600891 (2017).
68 Sluijs, A. *et al.* Environmental precursors to rapid light carbon injection at the Palaeocene/Eocene boundary. *Nature* **450**, 1218-1221 (2007).
69 Thomas, D. J., Zachos, J. C., Bralower, T. J., Thomas, E. & Bohaty, S. Warming the fuel for the fire: Evidence for the thermal dissociation of methane hydrate during the Paleocene-Eocene thermal maximum. *Geology* **30**, 1067-1070 (2002).
70 Cramwinckel, M. J. *et al.* Synchronous tropical and polar temperature evolution in the Eocene. *Nature* **559**, 382-386 (2018).
71 Tripati, A. K. *et al.* Tropical sea-surface temperature reconstruction for the early Paleogene using Mg/Ca ratios of planktonic foraminifera. *Paleoceanography* **18** (2003).
72 Inglis, G. N. *et al.* Descent toward the Icehouse: Eocene sea surface cooling inferred from GDGT distributions. *Paleoceanography* **30**, 1000-1020 (2015).
73 Bijl, P. K. *et al.* Eocene cooling linked to early flow across the Tasmanian Gateway. *Proc. Natl. Acad. Sci. USA* **110**, 9645-9650 (2013).
74 Wade, B. S. *et al.* Multiproxy record of abrupt sea-surface cooling across the Eocene-Oligocene transition in the Gulf of Mexico. *Geology* **40**, 159-162 (2012).
75 Pearson, P. N. *et al.* Extinction and environmental change across the Eocene-Oligocene boundary in Tanzania. *Geology* **36**, 179-182 (2008).
76 Liu, Z. *et al.* Global Cooling During the Eocene-Oligocene Climate Transition. *Science* **323**, 1187-1190 (2009).
77 Liu, Z. *et al.* Transient temperature asymmetry between hemispheres in the Palaeogene Atlantic Ocean. *Nat. Geosci.* **11**, 656-660 (2018).
78 O’Brien, C. L. *et al.* The enigma of Oligocene climate and global surface temperature evolution. *Proc. Natl. Acad. Sci. USA* **117**, 25302-25309 (2020).
79 Guitián, J. *et al.* Midlatitude temperature variations in the Oligocene to Early Miocene. *Paleoceanogr. Paleoclimatol.* **34**, 1328-1343 (2019).
80 Super, J. R. *et al.* North Atlantic temperature and $p\mathrm{CO_2}$ coupling in the early-middle Miocene. *Geology* **46**, 519-522 (2018).

81 Super, J. R. *et al.* Miocene evolution of North Atlantic sea surface temperature. *Paleoceanogr. Paleoclimatol.* **35**, e2019PA003748 (2020).
82 Methner, K. *et al.* Middle Miocene long-term continental temperature change in and out of pace with marine climate records. *Sci. Rep.* **10**, 7989 (2020).
83 Gómez, J. J. & Goy, A. Warming-driven mass extinction in the Early Toarcian (Early Jurassic) of northern and central Spain. Correlation with other time-equivalent European sections. *Palaeogeogr., Palaeoclimatol., Palaeoecol.* **306**, 176-195 (2011).
84 Retallack, G. J. *et al.* Eocene-Oligocene extinction and paleoclimatic change near Eugene, Oregon. *Geol. Soc. Am. Bull.* **116**, 817-839 (2004).
85 Hallam, A. & Wignall, P. B. *Mass Extinctions and their Aftermath*. (Oxford University Press, 1997).
86 Thomas, E. & Shackleton, N. J. The Paleocene-Eocene benthic foraminiferal extinction and stable isotope anomalies. *Geological Society London Special Publications* **101**, 401-441 (1996).
87 Gibbs, S. J., Bown, P. R., Sessa, J. A., Bralower, T. J. & Wilson, P. A. Nannoplankton extinction and origination across the Paleocene-Eocene thermal maximum. *Science* **314**, 1770-1773 (2006).
88 Longrich, N. R., Tokaryk, T. & Field, D. J. Mass extinction of birds at the Cretaceous–Paleogene (K–Pg) boundary. *Proc. Natl. Acad. Sci. USA* **108**, 15253-15257 (2011).
89 Longrich, N. R., Bhullar, B. S. & Gauthier, J. A. Mass extinction of lizards and snakes at the Cretaceous-Paleogene boundary. *Proc. Natl. Acad. Sci. USA* **109**, 21396-21401 (2012).
90 Wan, X., Wignall, P. B. & Zhao, W. The Cenomanian^Turonian extinction and oceanic anoxic event: evidence from southern Tibet. *Palaeogeogr., Palaeoclimatol., Palaeoecol.* **199**, 283-298 (2003).
91 Parente, M. *et al.* Stepwise extinction of larger foraminifers at the Cenomanian-Turonian boundary: A shallow-water perspective on nutrient fluctuations during Oceanic Anoxic Event 2 (Bonarelli Event). *Geology* **36**, 715-718 (2008).
92 Caswell, B. A., Coe, A. L. & Cohen, A. S. New range data for marine invertebrate species across the early Toarcian (Early Jurassic) mass extinction. *J. Geol. Soc. Lond.* **166**, 859-872 (2009).
93 Jiang, S., Song, H., Kemp, D. B., Dai, X. & Liu, X. Two pulses of extinction of larger benthic foraminifera during the Pliensbachian-Toarcian and early Toarcian environmental crises. *Palaeogeogr., Palaeoclimatol., Palaeoecol.* **560**, 109998 (2020).
94 Dal Corso, J. *et al.* Extinction and dawn of the modern world in the Carnian (Late Triassic). *Sci. Adv.* **6**, eaba0099 (2020).
95 Wignall, P. B. & Atkinson, J. W. A two-phase end-Triassic mass extinction. *Earth-Sci. Rev.* **208**, 103282 (2020).
96 Shen, S. *et al.* Calibrating the End-Permian Mass Extinction. *Science* **334**, 1367-1372 (2011).
97 Song, H., Wignall, P. B., Tong, J. & Yin, H. Two pulses of extinction during the Permian-Triassic crisis. *Nat. Geosci.* **6**, 52-56 (2013).
98 Benton, M. J. & Newell, A. J. Impacts of global warming on Permo-Triassic terrestrial ecosystems. *Gondwana Res.* **25**, 1308-1337 (2014).
99 Stanley, S. M. & Yang, X. A double mass extinction at the end of the Paleozoic Era. *Science* **266**, 1340-1344 (1994).
100 Brayard, A. *et al.* Good genes and good luck: Ammonoid diversity and the end-Permian mass extinction. *Science* **325**, 1118-1121 (2009).
101 Bond, D. *et al.* The mid-Capitanian (Middle Permian) mass extinction and carbon isotope record of South China. *Palaeogeogr., Palaeoclimatol., Palaeoecol.* **292**, 282-294 (2010).
102 Kaiser, S. I., Aretz, M. & Becker, R. T. The global Hangenberg Crisis (Devonian–Carboniferous transition): review of a first-order mass extinction. *Geological Society, London, Special Publications* **423**, 387-437 (2016).
103 Sallan, L. & Coates, M. I. End-Devonian extinction and a bottleneck in the early evolution of modern jawed

vertebrates. *Proc. Natl. Acad. Sci. USA* **107**, 10131-10135 (2010).

104 Myrow, P. M. *et al.* High-precision U–Pb age and duration of the latest Devonian (Famennian) Hangenberg event, and its implications. *Terra Nova* **26**, 222-229 (2014).

105 Rong, J. *et al.* The latest Ordovician Hirnantian brachiopod faunas: New global insights. *Earth-Sci. Rev.* **208**, 103280 (2020).
