# Supplementary material for: Thresholds of temperature change for mass extinctions
Source: Nat Commun. 2021 Aug 4;12:4694. doi: 10.1038/s41467-021-25019-2 (PMC8338942; doi:10.1038/s41467-021-25019-2)
Supplement: Supplementary file 3 — Description of Additional Supplementary Information [file 41467_2021_25019_MOESM3_ESM.docx]

**Description of Additional Supplementary Files**

File Name: Source Data

Description: Temperature and extinction data used to construct Figs. 1-4 of main text.

File Name: Supplementary Data 1

Description: Temperature database for calculating the magnitudes and rates of temperature change. This file is available at <https://github.com/haijunsong/Thresholds-of-temperature>.
