# Supplementary material for: Thresholds of temperature change for mass extinctions
Source: Nat Commun. 2021 Aug 4;12:4694. doi: 10.1038/s41467-021-25019-2 (PMC8338942; doi:10.1038/s41467-021-25019-2)
Supplement: Supplementary file 5 — Reporting Summary [file 41467_2021_25019_MOESM5_ESM.pdf]

nature research

Corresponding author(s): Haijun Song
Last updated by author(s): 24-6-2021

Reporting Summary

Nature Research wishes to improve the reproducibility of the work that we publish. This form provides structure for consistency and transparency in reporting. For further information on Nature Research policies, see our Editorial Policies and the Editorial Policy Checklist.

Statistics

For all statistical analyses, confirm that the following items are present in the figure legend, table legend, main text, or Methods section.

n/a	Confirmed	
☐	☒	The exact sample size (n) for each experimental group/condition, given as a discrete number and unit of measurement
☒	☐	A statement on whether measurements were taken from distinct samples or whether the same sample was measured repeatedly
☐	☒	The statistical test(s) used AND whether they are one- or two-sided<br>*Only common tests should be described solely by name; describe more complex techniques in the Methods section.*
☒	☐	A description of all covariates tested
☐	☒	A description of any assumptions or corrections, such as tests of normality and adjustment for multiple comparisons
☐	☒	A full description of the statistical parameters including central tendency (e.g. means) or other basic estimates (e.g. regression coefficient) AND variation (e.g. standard deviation) or associated estimates of uncertainty (e.g. confidence intervals)
☐	☒	For null hypothesis testing, the test statistic (e.g. F, t, r) with confidence intervals, effect sizes, degrees of freedom and P value noted<br>*Give P values as exact values whenever suitable.*
☐	☒	For Bayesian analysis, information on the choice of priors and Markov chain Monte Carlo settings
☒	☐	For hierarchical and complex designs, identification of the appropriate level for tests and full reporting of outcomes
☐	☒	Estimates of effect sizes (e.g. Cohen's d, Pearson's r), indicating how they were calculated

Our web collection on statistics for biologists contains articles on many of the points above.

Software and code

Policy information about availability of computer code

Data collection	Excel was used in the data collection process.
Data analysis	Data were input and plotted in Excel (2013). Correlation analysis was performed in SPSS 17.0. Paleolatitudes were reconstructed using PointTracker v7 rotation files. Extinction rate and temperature estimate were performed in R. Oxygen isotope values from carbonate fossils (i.e., planktonic foraminifera, brachiopod, oyster, and belemnite) are converted to SSTs using the BAYFOX Bayesian model (https://github.com/jesstierney/bayfoxm). All scripts used to conduct analyses are available at https://github.com/haijunsong/Thresholds-of-temperature.

For manuscripts utilizing custom algorithms or software that are central to the research but not yet described in published literature, software must be made available to editors and reviewers. We strongly encourage code deposition in a community repository (e.g. GitHub). See the Nature Research guidelines for submitting code & software for further information.

Data

Policy information about availability of data

All manuscripts must include a data availability statement. This statement should provide the following information, where applicable:

- Accession codes, unique identifiers, or web links for publicly available datasets
- A list of figures that have associated raw data
- A description of any restrictions on data availability

All temperature data are available in the supplementary information. Supplementary Database 1 is available at https://github.com/haijunsong/Thresholds-of-temperature. Fossil occurrence data are available from the Paleobiology Database (http://www.paleobiodb.org).

Field-specific reporting

Please select the one below that is the best fit for your research. If you are not sure, read the appropriate sections before making your selection.

☐ Life sciences ☐ Behavioural & social sciences ☒ Ecological, evolutionary & environmental sciences

For a reference copy of the document with all sections, see nature.com/documents/nr-reporting-summary-flat.pdf

Ecological, evolutionary & environmental sciences study design

All studies must disclose on these points even when the disclosure is negative.

Study description	We analyzed magnitudes and rates of temperature change and marine fossil biodiversity through 45 time intervals spanning the past 450 million years. Phanerozoic paleotemperature data mainly come from paleothermometry methods including oxygen isotope (δ18O), clumped isotope (Δ47), organic geochemical proxy (TEX86). We show the calculated magnitudes and rates of temperature for the 45 time bins in Table S1 and original data in Database S1.
Research sample	Oxygen isotope values were derived from carbonate fossils (i.e., plankton foraminifers, brachiopods, oysters, and belemnites) and phosphate fossils (conodonts). Clumped isotope measurements were derived from rugose corals, brachiopods, bryozoans, and trilobites. TEX86 values were derived from bulk rock samples. Gap-filler and three-timer extinction rates of marine animals were calculated using data from the Paleobiology Database (PBDB, http://paleobiodb.org), which was downloaded on 4 January 2021. The fossil dataset includes all metazoans except for Arachnida, Insecta, Ostracoda, and Tetrapoda and consists of 850,840 fossil occurrences of 37,134 genera. These four groups that are excluded were not used because many of them (Arachnida, Insecta, and Tetrapoda) are terrestrial and appear in marine strata. Ostracoda also have a record in terrestrial rocks.
Sampling strategy	The temperature database is composed of the most significant warming/cooling events in 45 time intervals from the late Ordovician (445 Ma) to early Miocene (16 Ma). The time intervals are consistent with the time bins used to compute biodiversity and evolutionary rates, and are defined by one or several neighboring geologic stages with roughly uniform durations (averaging 9.71 Myr). We focused on time intervals with well-studied and reasonably high-resolution geochemistry data. Time bins with only low-resolution paleotemperature data (i.e., < 2 measurements per million years) are excluded, e.g., all bins in the Cambrian and most bins in the Ordovician.
Data collection	All data were collected from published papers. Fossil data were downloaded from http://paleobiodb.org by Xu Dai. Oxygen isotope (δ18O), clumped isotope (Δ47), and organic geochemistry (TEX86) data were collected using Excel from published literatures by Haijun Song.
Timing and spatial scale	Most paleotemperature data are sea surface temperatures (SST) from tropical and subtropical regions (between 40°N and 40°S). Only one collection is from a mid-latitude region with a paleolatitude of 42.71°N.
Data exclusions	Data that were likely affected by diagenesis or local effects (e.g., δ18O data from carbonate fossil shells with Mn> 250 ppm and Sr < 400 ppm, TEX86 data with BIT >0.4, Methane Index (MI) >0.5, delta-Ring Index (ΔRI) > 0.3, %GDGT-0 >67%, and/or fCren′:Cren′ + Cren >0.25) were removed from the database. In addition, temperature data from high paleo-latitude regions (>40°) were excluded. Time bins with only low-resolution paleotemperature data (i.e., < 2 measurements/Myr) are excluded. The exclusion criteria were pre-established.
Reproducibility	This is not an experimental study so experimental replication was not attempted. All data used in our analysis are from published papers and publicly available. The results can be reproduced using these data and methods.
Randomization	No randomization was required. Our study was not experimental, but based on published paleo-temperature values and biodiversity.
Blinding	Our study was not experimental and so blinding is not relevent

Did the study involve field work? ☐ Yes ☒ No

Reporting for specific materials, systems and methods

We require information from authors about some types of materials, experimental systems and methods used in many studies. Here, indicate whether each material, system or method listed is relevant to your study. If you are not sure if a list item applies to your research, read the appropriate section before selecting a response.

Materials & experimental systems

n/a	Involved in the study
☒	☐ Antibodies
☒	☐ Eukaryotic cell lines
☒	☐ Palaeontology and archaeology
☒	☐ Animals and other organisms
☒	☐ Human research participants
☒	☐ Clinical data
☒	☐ Dual use research of concern

Methods

n/a	Involved in the study
☒	☐ ChIP-seq
☒	☐ Flow cytometry
☒	☐ MRI-based neuroimaging
